# Delivering Multidisciplinary Cardiac Care in Conflict Zones: A Cardiology Mission to Homs, Syria

**DOI:** 10.1016/j.jscai.2025.104057

**Published:** 2025-12-30

**Authors:** Mohammad Al-Akchar, Rafel S. El-Atassi, Abdulah Alrifai, Ghiyath A. Al-Tabbal, Sameh Sayfo

**Affiliations:** a Southern Illinois University, Springfield, Illinois; b Mercy Health, Toledo, Ohio; c Advocate Heart Institute, Hazel Crest, Illinois; d Wesley Healthcare, Wichita, Kansas; e Department of Cardiovascular Medicine, Baylor Scott & White The Heart Hospital − Plano, Plano, Texas; f Texas A&M University School of Medicine, Bryan, Texas

**Keywords:** cardiology, conflict zones, medical missions, Syria

## Introduction

In April 2025, a multidisciplinary cardiology team conducted a medical mission to Homs, Syria. The mission was conducted over a 10-day period and aimed to address significant gaps in cardiovascular care within a conflict zone. The team delivered advanced cardiac services, including inpatient and outpatient consultations, echocardiography, percutaneous coronary interventions (PCI), pacemaker and implantable cardioverter-defibrillator implantation, and electrophysiology-guided care. The mission team comprised 60 health care professionals, including 6 cardiologists, anesthesiologists, intensivists, general physicians, and logistics personnel, enabling a truly multidisciplinary approach.

This initiative was critical, given that more than 14 years of conflict have severely disrupted Syria’s health care infrastructure, resulting in inadequate access to specialty care. Cardiovascular disease remains a leading cause of mortality in the Middle East,^1^ even in conflict-affected regions, necessitating targeted interventions to bridge care gaps.

## Health care challenges in Syria

Syria’s health care sector has endured substantial strain from prolonged conflict, including damaged infrastructure, shortages of equipment and medications, and disrupted training pathways for local physicians. The burden of cardiovascular disease—including coronary artery disease, heart failure, arrhythmias, and valvular pathologies—remains high despite limited access to advanced diagnostic and interventional therapies. Medical missions, therefore, serve dual roles—providing immediate care and contributing to long-term capacity building through local training and infrastructure development.^2^

## Mission overview

The mission team included 6 cardiologists with subspecialties in general cardiology, interventional cardiology, and electrophysiology. They collaborated closely with local cardiology fellows and specialists at 2 hospitals—1 governmental (Ibn Alwaleed) and 1 charitable (Albir). The invasive procedures were performed at Ibn Alwaleed Hospital, a 75-bed hospital with 1 catheterization laboratory, 12 intensive care unit beds, and no surgical backup. Multidisciplinary participation from intensivists, general physicians, and nurses enabled the team to address a broader spectrum of cardiovascular care needs.

## Patient selection and pre-mission planning

Patient selection was coordinated in partnership with local physicians prior to the team’s arrival. Virtual planning sessions involved reviewing imaging, selecting procedural candidates, and confirming equipment availability.

During the 10-day mission, the team conducted 65 unique outpatient consultations and managed 55 unique inpatients, averaging 15 inpatient rounds per day. Patients presented with a wide range of pathologies, including coronary artery disease, decompensated heart failure, rheumatic valvular disease, and arrhythmias. Among these, approximately 70% of patients had significant changes to their management, including coronary revascularization, device implantation (pacemakers and defibrillators), or modification of medical therapy.

## Cardiology services provided

### General cardiology and imaging

The team performed comprehensive inpatient and outpatient consultations supported by transthoracic and transesophageal echocardiography. Despite limitations, including the absence of imaging stress testing, lack of 3D imaging and offline interpretation software, and outdated sterilization techniques, local fellows were trained to perform echocardiography in alignment with the American Society of Echocardiography guidelines, improving diagnostic accuracy and reporting consistency.

### Interventional cardiology services

A total of 35 diagnostic angiograms and 9 PCI procedures were performed, including bifurcation stenting and calcified lesion PCI. All procedures were successfully performed without complications except for 1 ST-elevation myocardial infarction case with no-reflow phenomena. A patient was treated with intravenous glycoprotein IIb/IIIa receptor inhibitor, but unfortunately, he ended up with severe ischemic cardiomyopathy with an ejection fraction of 30%. Although protective equipment was partially available at the hospitals, the mission team provided additional on-site training on safe radiation practices for fellows and catheterization laboratory staff. The training focused on the appropriate utilization of shields, understanding the importance of minimizing radiation time and cineangiography time, depending more on fluoroscopy, and adhering to the as low as reasonably achievable (ALARA) principle. We also provided fellows and trainees in the laboratory with radiation protection goggles. Future missions plan to provide dosimetry badges to enhance long-term safety.

### Electrophysiology services

Pacemaker and implantable cardioverter-defibrillator implantation procedures were performed for patients with sick sinus syndrome, complete heart block, and heart failure with reduced ejection fraction. Lead aprons and thyroid shields were used where available, and the mission team delivered focused education on minimizing fluoroscopy exposure for local fellows. Electrophysiology studies and mapping systems were unavailable, underscoring ongoing infrastructure needs. All electrophysiology procedures were performed without complications, such as no pocket infection, bleeding, or mortality.

## Establishing a valve clinic

One of the mission’s most impactful achievements was establishing Homs’ first multidisciplinary valve clinic. The clinic comprised clinical cardiologists, interventional cardiologists, and a cardiothoracic surgeon, enabling collaborative evaluation of 20 complex valvular cases.

After completing the mission, the valve clinic operations evolved into serial consultations (depending on ongoing mission members), weekly virtual heart-team discussions, and combined review of imaging and test results.

The valve clinic has continued to operate virtually through secure WhatsApp-based data sharing between visiting and local physicians. Patients requiring surgical or transcatheter interventions are batched and scheduled during future missions, ensuring continuity of care despite resource constraints. Currently, most valve patients are treated surgically if they are able to afford it; if the patient is high risk, then only medical therapy is usually provided. The first valve clinic consisted of an interventional cardiologist, a cardiothoracic surgeon, and an imaging cardiologist, and the patients were evaluated by the team at the same time, and when a transesophageal echocardiogram was deemed necessary, it was performed by the echocardiographer. Other non-physician members of the team included 3 nurses, who were responsible for patient registration, record collection, data entry, and assisting with imaging (transthoracic and transesophageal echo). No dedicated coordinator was identified at that time; however, a senior fellow assumed the role.

Among the patients who were evaluated, 3 of them received TAVR during the next mission, which took place in the month of June, 1 received ASD closure, 3 received a coronary angiogram and an intervention, 3 received surgical intervention, and the rest received medical therapy, with one of them requiring hospital admission for acute heart failure due to severe mitral stenosis. The PCI procedures were performed at Ibn Alwaleed Hospital, and structural procedures and surgical interventions took place during the following mission at Alzira’a hospital, a charitable hospital that was built 5 years ago by the Syrian American Medical Society.

## Role of local physicians and trainees

Local cardiologists and fellows were involved both prior to arrival—by identifying potential procedural candidates—and during the mission, when they participated directly in patient selection, procedural planning, and bedside teaching.

Hands-on training was provided through bedside teaching, procedural assistance, and structured lectures. Although local fellows are not yet independently performing PCI or device implantations, their echocardiography skills have improved significantly, now aligning with the American Society of Echocardiography standards. This foundation supports sustainable skill-building and strengthens local capacity.

## Logistical challenges and equipment procurement

Equipment shortages required creative solutions. Stents, pacemakers, and procedural supplies were transported across the border with support from partner humanitarian organizations. Medical equipment was labeled as humanitarian aid to expedite clearance, and select consumables were procured locally whenever possible. Collaboration with local hospital teams was essential to navigating infrastructure constraints.

## Cultural and ethical considerations

The mission respected local cultural norms, where health care decisions are often made collectively by families. Informed consent discussions frequently involved multiple relatives, and shared decision-making frameworks were adopted to ensure patient autonomy while honoring local practices. Ethical standards were upheld consistently, prioritizing transparency, dignity, and cultural sensitivity.

## Postmission sustainability and follow-up

Postmission follow-up has been virtual, with ongoing case discussions between local physicians and the visiting team. The April mission played a foundational role in future missions. Since then, 3 additional missions (2 from the USA and 1 from Germany) have occurred. The plan is to scale the multidisciplinary clinic experience to other cities. A unified effort has launched not only a full academic clinical curriculum for fellows but also a research curriculum for medical students. The communication between the mission team and local physicians and trainees continued after the mission ended, during which many cases were discussed, and a second opinion was obtained virtually. The multidisciplinary clinic continued in virtual form, and the plan is for more in-person clinics during future missions.

## Recommendations for future missions

Future missions should:

1. Continue integrating virtual case discussions to support continuity of care.
2. Expand hands-on training for fellows.
3. Strengthen radiation safety practices through structured on-site education.
4. Develop collaborative infrastructure, such as shared imaging archives, to streamline decision-making.
5. Sustain partnerships with humanitarian organizations to secure necessary supplies and equipment.

## Conclusion

This 10-day multidisciplinary mission to Homs, Syria, highlights the value of collaborative outreach in delivering advanced cardiovascular care in resource-limited, conflict-affected settings. By providing procedural interventions, training local providers, and establishing a sustainable valve clinic, the mission addressed immediate clinical needs while laying the groundwork for long-term capacity building. Continued partnerships, virtual consultations, and iterative missions will be essential for improving cardiovascular outcomes in similar regions.

## Declaration of competing interest

The authors declared no potential conflicts of interest with respect to the research, authorship, and/or publication of this article.
